# Purification and biochemical characterization of SM14est, a PET-hydrolyzing enzyme from the marine sponge-derived *Streptomyces* sp. SM14

**DOI:** 10.3389/fmicb.2023.1170880

**Published:** 2023-05-12

**Authors:** Clodagh M. Carr, Malene B. Keller, Bijoya Paul, Sune W. Schubert, Kristine S. R. Clausen, Kenneth Jensen, David J. Clarke, Peter Westh, Alan D. W. Dobson

**Affiliations:** ^1^School of Microbiology, University College Cork, Cork, Ireland; ^2^SSPC-SFI Research Centre for Pharmaceuticals, University College Cork, Cork, Ireland; ^3^Department of Biotechnology and Biomedicine, Technical University of Denmark, Lyngby, Denmark; ^4^Novozymes A/S, Lyngby, Denmark; ^5^APC Microbiome Ireland, University College Cork, Cork, Ireland; ^6^Environmental Research Institute, University College Cork, Cork, Ireland

**Keywords:** plastic, marine, PETase, biorecycling, bioremediation

## Abstract

The successful enzymatic degradation of polyester substrates has fueled worldwide investigation into the treatment of plastic waste using bio-based processes. Within this realm, marine-associated microorganisms have emerged as a promising source of polyester-degrading enzymes. In this work, we describe the hydrolysis of the synthetic polymer PET by SM14est, a polyesterase which was previously identified from *Streptomyces* sp. SM14, an isolate of the marine sponge *Haliclona simulans*. The PET hydrolase activity of purified SM14est was assessed using a suspension-based assay and subsequent analysis of reaction products by UV-spectrophotometry and RP-HPLC. SM14est displayed a preference for high salt conditions, with activity significantly increasing at sodium chloride concentrations from 100 mM up to 1,000 mM. The initial rate of PET hydrolysis by SM14est was determined to be 0.004 s^−1^ at 45°C, which was increased by 5-fold to 0.02 s^−1^ upon addition of 500 mM sodium chloride. Sequence alignment and structural comparison with known PET hydrolases, including the marine halophile PET6, and the highly efficient, thermophilic PHL7, revealed conserved features of interest. Based on this work, SM14est emerges as a useful enzyme that is more similar to key players in the area of PET hydrolysis, like PHL7 and IsPETase, than it is to its marine counterparts. Salt-tolerant polyesterases such as SM14est are potentially valuable in the biological degradation of plastic particles that readily contaminate marine ecosystems and industrial wastewaters.

## 1. Introduction

The *Streptomyces* genus has proven to be a worthwhile source of secondary metabolites, ranging from antimicrobial to cytotoxic compounds, with human health needs being the primary driver of the discovery efforts for these bioactive products (Lacey and Rutledge, 2022). As with other actinomycetes, *Streptomyces* species are also important producers of industrially relevant enzymes (Kumar et al., 2020), although perhaps they have not yet been as well-explored in this regard to date. The last decade has seen a notable shift away from terrestrial ecosystems toward the marine environment as a source of microorganisms harboring novel biological activities (Dharmaraj, 2010; Yang et al., 2020; Olaniyan and Adetunji, 2021). Nevertheless, given the vast and varied nature of the Earth's oceans, we are still in the early stages of the quest to describe the full potential of the marine microbiome (Abreu et al., 2022). In particular, marine invertebrates such as sponges have been shown to represent a hotspot of microbial biodiversity and activity, brought about by the mutualistic interactions that exist between microbial symbionts and the host sponge, which have the potential to be sustainably exploited for biotechnological purposes (Amelia et al., 2022).

Marine sponges harbor diverse communities of bacteria, archaea, and unicellular eukaryotes, which inhabit sponge tissues at densities of over 10^9^ cells/cm^3^ and contribute to as much as 35% of the total sponge biomass (Hentschel et al., 2012). Sponge-associated microbes are known to be quite prolific producers of natural products, as well as extracellular enzymes, which are believed to contribute positively to the metabolism and overall health of their sea sponge host (Taylor et al., 2007; de Oliveira et al., 2020; Nnaji et al., 2022). Such enzymes are often hydrolase family members and are likely to be produced in an attempt to degrade the wide variety of substrates present in the large quantities of seawater being filtered by the sponge, providing both symbiotic partners with a source of energy and nutrients (Wang, 2006; Pita et al., 2018; Birolli et al., 2019). In the marine environment, heterotrophic microbes are responsible for the biogeochemical cycling of dissolved organic carbon, an abundant reservoir comprising thousands of distinct chemical structures. However, dissolved organic carbon compounds are often quite complex and recalcitrant to degradation, thus requiring adaptable enzymes for their utilization as substrates (Wagner et al., 2020). Marine microbial hydrolases must also withstand the changeable and challenging conditions associated with ocean waters, further supporting the potential applicability of these resilient enzymes across a range of new biotech products and services (Sana, 2015).

**Figure 1:**
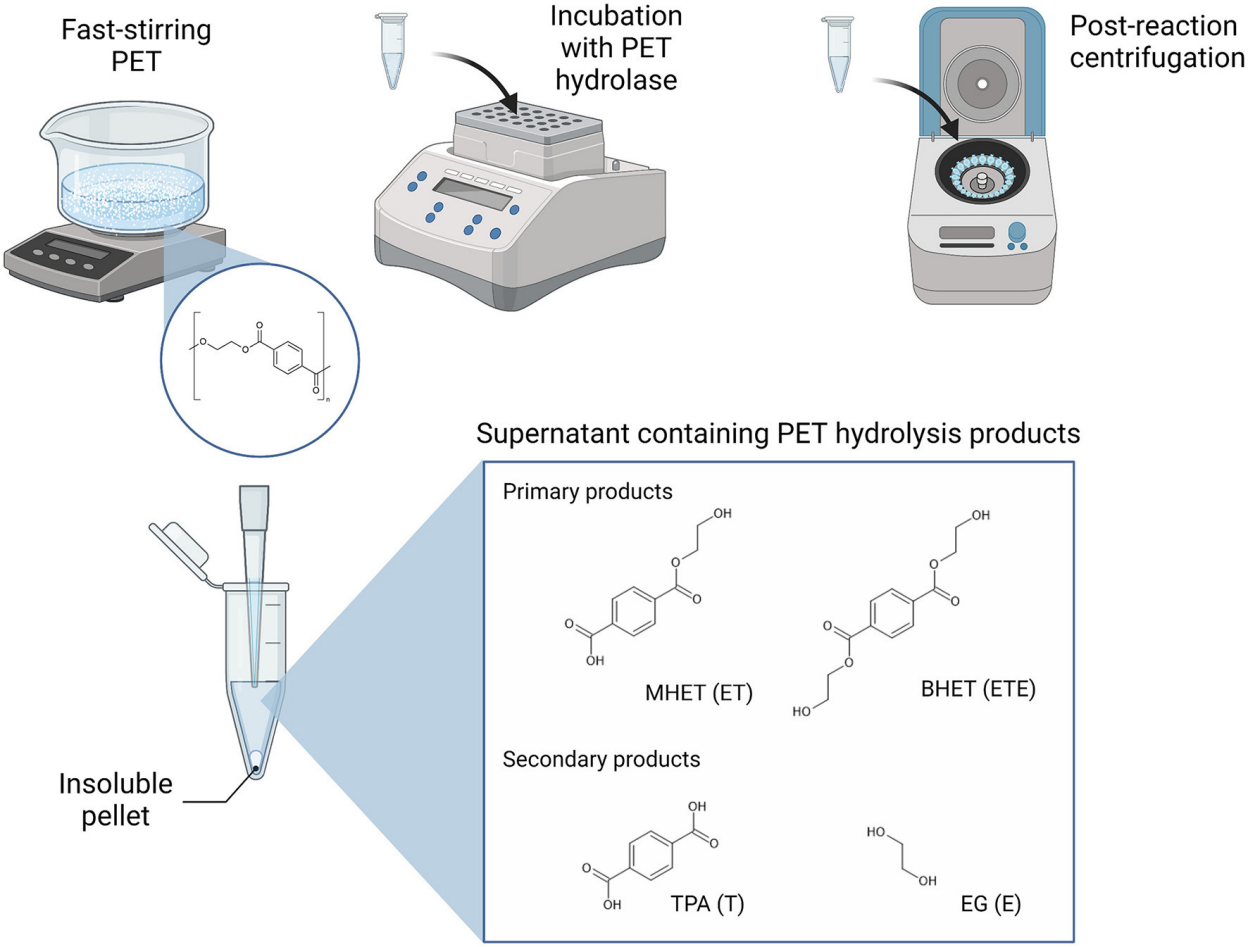
Experimental setup for the detection of the soluble products of PET hydrolysis. Polymeric PET substrate is suspended in the reaction buffer and homogeneity is maintained with fast stirring. The PET substrate and PET hydrolase of interest are incubated with continuous shaking under the selected temperature and time parameters. Upon successful PET hydrolysis, the soluble fraction will include the shortest oligomers, MHET and BHET, and the simplest monomeric units, TPA and EG, which are separated from insoluble PET material by centrifugation. The aromatic ring-containing hydrolysis products may be detected from the supernatant, for example, by UV-spectrophotometry alone or coupled to RP-HPLC for separation of individual products.

PET hydrolase enzymes have, for the past number of years, been the focus of extensive research and engineering efforts to support the biological recycling of polyester plastics, with increasing success since the identification of TfH, a PET-hydrolyzing enzyme from *Thermobifida fusca*, in 2005 (Wei et al., 2022). LC-cutinase (LCC) from a leaf compost-derived metagenome has represented a highly active benchmark PET hydrolase (Andler et al., 2022; Wei et al., 2022). The LCC variant, ICCG, which can achieve 90% degradation of pre-treated post-consumer PET within 10 h, has even been implemented as a catalyst in pilot-scale PET bio-recycling (Tournier et al., 2020). Most recently, HotPETase and FAST-PETase, which are thermostable variants of IsPETase from the mesophilic bacterium *Ideonella sakaiensis*, have been engineered to match the activities of LCC and ICCG (Bell et al., 2022; Lu et al., 2022). During enzymatic hydrolysis, the polyethylene terephthalate (PET) polymer is primarily converted into the intermediate products MHET [mono(2-hydroxyethyl terepththalate)] and [bis(2-hydroxyethyl terepthalate)], which may then be further degraded into the monomeric subunits terephthalic acid (TPA) and ethylene glycol (EG; Figure 1). TPA and EG can also be further abbreviated to T and E, respectively, which allows for short-hand notation of the PET breakdown products that reflects their composition and length, for example, where ET is MHET and ETE is BHET (Schubert et al., 2023).

Within the realm of enzymatic plastic degradation, several groups have investigated marine-derived microbes for polyester hydrolases, particularly those capable of breaking the ester bonds that link the subunits of the synthetic polyester PET (Blázquez-Sánchez et al., 2022; Meyer Cifuentes et al., 2022; Weigert et al., 2022). PET waste is widely distributed in the marine environment, both in macro and microplastic forms, thereby posing a significant threat to marine life and ultimately to humans (Nabi et al., 2022). While it is interesting to consider the enzymatic degradation of PET by marine microorganisms, both from an ecological and biotechnological perspective, it is not yet well-understood whether this capacity has evolved specifically in response to plastic pollution in our oceans or if this is among the many unconventional side reactions that hydrolases can facilitate when deprived of simpler substrates that are more easily metabolized (Barzkar et al., 2021; Dittmar et al., 2021; Jiménez et al., 2022).

Our group had previously identified SM14est, a polyesterase from the marine-sponge derived *Streptomyces* sp. SM14, during the mining of 52 *Streptomyces* genome sequences for PET hydrolase-encoding genes. Polycaprolactone (PCL) was used to confirm polyesterase activity of the heterologously expressed enzyme in an agar-based plate clearing assay (Almeida et al., 2019b). Although petroleum-based PCL and PET are both employed to produce semi-crystalline thermoplastics, the biodegradable polycaprolactone only serves as a model substrate for the more complex PET, which is an aliphatic-aromatic co-polyester (Niaounakis, 2017; Molitor et al., 2020). Based on computational characterization and molecular docking with BHET, the ester of PET monomeric units EG and TPA, hydrolytic activity toward PET was predicted but not confirmed (Almeida et al., 2019b).

Here we present biochemical and kinetic data for SM14est, obtained using a suspension-based assay that allows for the detection of PET hydrolysis products. A *Bacillus subtilis* heterologous host was employed to produce and secrete recombinant SM14est, which was his-tagged and subsequently affinity purified. The purified SM14est was found to hydrolyze PET following incubation at 45°C in sodium phosphate buffer (pH 8), with products measured by UV-spectrophotometry. This activity toward PET was enhanced with increasing concentrations of sodium chloride (100–1,000 mM) and further progress curve analysis revealed a 5-fold increase in the initial rate of PET hydrolysis when sodium chloride (500 mM) was included in the reaction.

## 2. Materials and methods

### 2.1. Enzyme expression and purification

SM14est (Genbank: DAC80635.1) was codon optimized for heterologous expression in *Bacillus subtilis* and produced as previously described (Jensen et al., 2010), with modification during construct design such that the native SM14est signal peptide was replaced by AmyL (FJ556804.1) from *B. licheniformis* and a hexa histidine-tag was incorporated at the C-terminus of the protein. The recombinant SM14est protein (29 kDa) was purified by Ni-affinity chromatography on a HisTrap FF 5 mL column (Cytiva) using the ÄKTA™ Pure system. A peristaltic pump (MiniPuls 3, Gilson^®^) was used for the initial column wash and loading steps. The purified protein was desalted using Spectra/Por^®^ dialysis membrane (MWCO 12–14 kDa). The molar extinction coefficient of 39,880 M^−1^ cm^−1^ was predicted by ExPASy-ProtParam and was used to calculate the concentration of purified enzyme from the absorbance measured at 280 nm. The SM14est sample was visualized following SDS-PAGE of 2.5 μg of purified protein on a Tris-Glycine 4–20% gel (Bio-Rad Laboratories). Thermal denaturation experiments were conducted in triplicate with 10 μM of enzyme and analyzed by nano differential scanning fluorimetry (nanoDFS) using a temperature slope of 2°C min^−1^ (Prometheus NT.48, Nano Temper).

### 2.2. Suspension-based PET activity assay

A suspension-based assay (Bååth et al., 2020) was used to investigate the hydrolytic activity of SM14est toward semi-crystalline PET powder reported to have >40% crystallinity with a particle size of <300 μM (Goodfellow Co, product number ES306031/1). Depending on the required assay setup, this microPET powder was suspended either in the selected buffer or Milli-Q water and pipetted while under vigorous stirring. The PET substrate suspension was transferred to low-binding microplates (Greiner Bio-One™ 655901) or microtubes (Sarstedt 72.706.600), followed by additional buffer, and finally the purified enzyme.

The reactions were performed in triplicate, with samples incubated in a thermomixer (Eppendorf) at 1,100 rpm. Based on preliminary results, assays were typically carried out at 45°C using 0.5 μM enzyme, 10 g L^−1^ PET, and 50 mM sodium phosphate buffer pH 8 in a final reaction volume of 250 μL. Samples were centrifuged to quench the reaction, then supernatants (100 μL) were transferred into a UV-transparent microplate (Corning) to measure soluble aromatic products of PET hydrolysis, such as TPA, MHET, and BHET, at absorbance 240 nm in a plate reader (Molecular Devices SpectraMax Paradigm). As previously described, products were quantified from the absorbance readings by comparing with standard curves for BHET and converting to BHET equivalents (μM), providing a platform for screening and initial characterization of potential PET hydrolase enzymes (Bååth et al., 2020). It should be noted that it is not possible to differentiate between the individual soluble products using this method, however, BHET has been previously employed as a standard to reflect the pooled absorbance of soluble products (Thomsen et al., 2022).

### 2.3. Progress curve analyses

Supernatant time-point samples were taken at *t* = 1, 3, 5, and 7 h post-incubation for measurement at 240 nm (as previously described). An additional 100 μL was retained from the remaining supernatant, treated with hydrochloric acid (2 μL, 5M) to quench the reaction, and stored at −20°C until further quantification using reversed-phase high-performance liquid chromatography (RP-HPLC; Thermo Scientific™, Vanquisher: Capital HPLC). Formic acid (7.5 mM) and acetonitrile (5% v/v) were used during the injection of samples (20 μL) at a ratio of 1:5 for 7.5 min, prior to elution using acetonitrile for 12.5 min, with a flow rate of 1 mL min^−1^ maintained at 40°C. The analytes were detected by UV-spectrophotometry at 240 nm and Chromeleon chromatography studio software (version 7.3.1) was used to perform peak analysis. The mono-aromatic products of PET hydrolysis were quantified against standards of TPA, MHET, BHET, while longer di- and tri-aromatic compounds were quantified against standards of TETE and TETET. All reactions were performed in duplicate and substrate blanks were included for the quantification of autohydrolysis.

### 2.4. Computational protein analyses

Protein sequences were aligned using T-COFFEE (Di Tommaso et al., 2011) in Expresso (structural alignment) mode, and outputs were visualized with ESPript 3.0 (Gouet et al., 1999). Alpha-Fold, which combines a deep learning-based algorithm with well-curated experimental knowledge from pre-existing structure and sequence databases, was used to model the SM14est protein as it is considered to be highly accurate with respect to atomic structure (Jumper et al., 2021). The Alpha-Fold-predicted structure for SM14est was verified as a good quality model based on the per-residue confidence scores (pLDDT) and the Predicted Aligned Error (PAE) plots (Supplementary Figure 1). The SM14est model was visualized in UCSF-Chimera and the MatchMaker tool was used to facilitate structural comparison with other PET hydrolases (Pettersen et al., 2004).

## 3. Results

### 3.1. Purification of SM14est and T_*m*_ analysis

**Figure 2:**
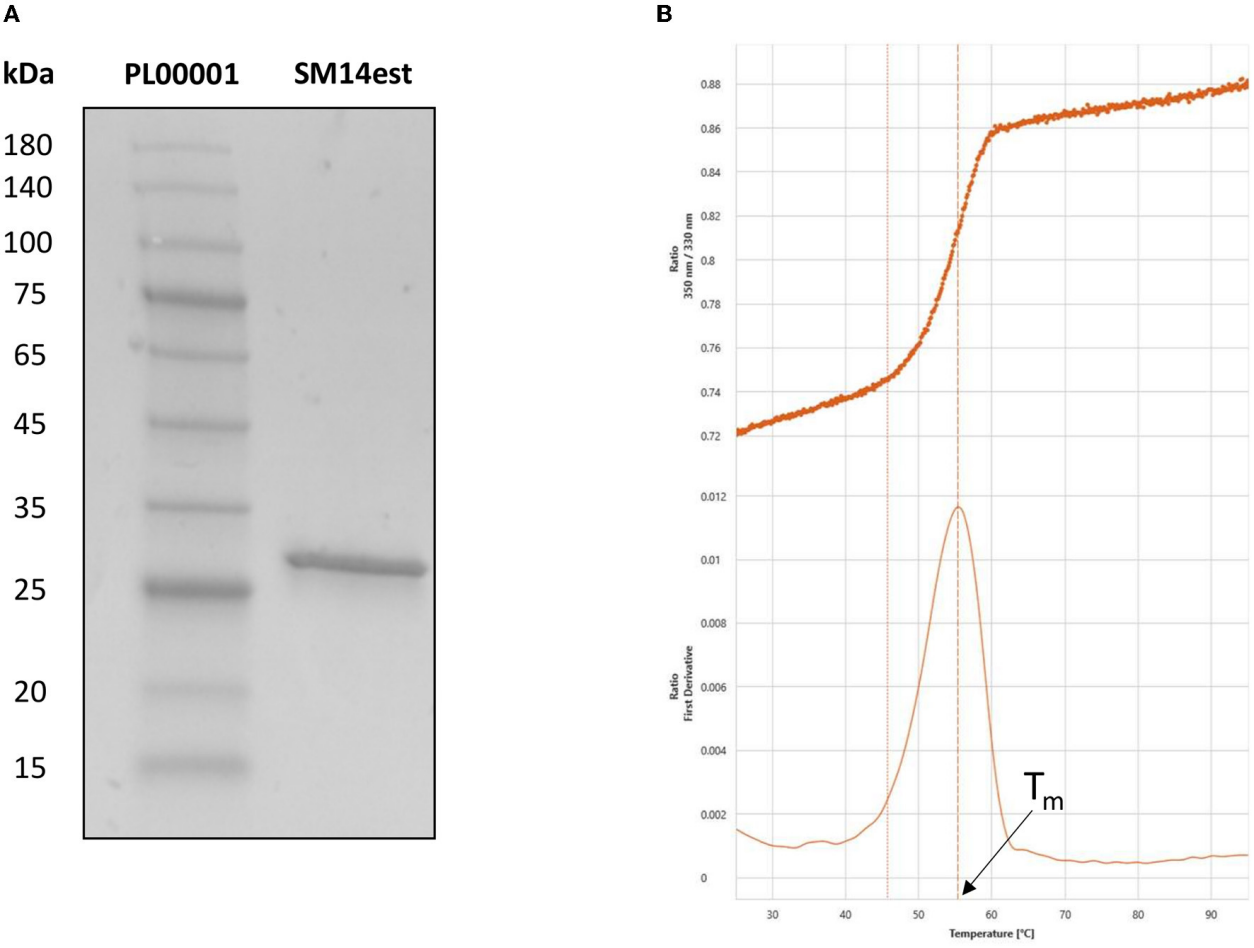
Post-purification analyses of SM14est by **(A)** SDS-PAGE, with pre-stained protein marker in lane 1 and SM14est in lane 2 and **(B)** NanoDFS, with the F350/330 ratio and its first derivative shown for T_m_ determination.

The expression and purification of SM14est was evaluated by SDS-PAGE analysis, which displayed a single band at around 29 kDa that corresponded to the predicted molecular weight of the recombinant SM14est protein (Figure 2A). Nano differential scanning fluorimetry was employed to study protein denaturation and the thermal transition midpoint (T_m_) of SM14est was subsequently determined to be 55°C in sodium phosphate buffer, pH 8 (Figure 2B). Here, the intrinsic fluorescence of aromatic amino acid residues, primarily tryptophan, was used to track protein unfolding by measuring and recording changes in the fluorescence intensity ratio (350:330 nm), yielding a value for T_m_ when plotted as a function of temperature (Wen et al., 2020). The inflection point and isolated peak observed following the nanoDFS scan further validated the purity of the SM14est protein sample, given that a mixed sample would have generated a linear F350/330 signal and multiple first derivative peaks.

### 3.2. Investigating the effect of temperature, salt, and pH on SM14est PET-hydrolyzing activity

**Figure 3:**
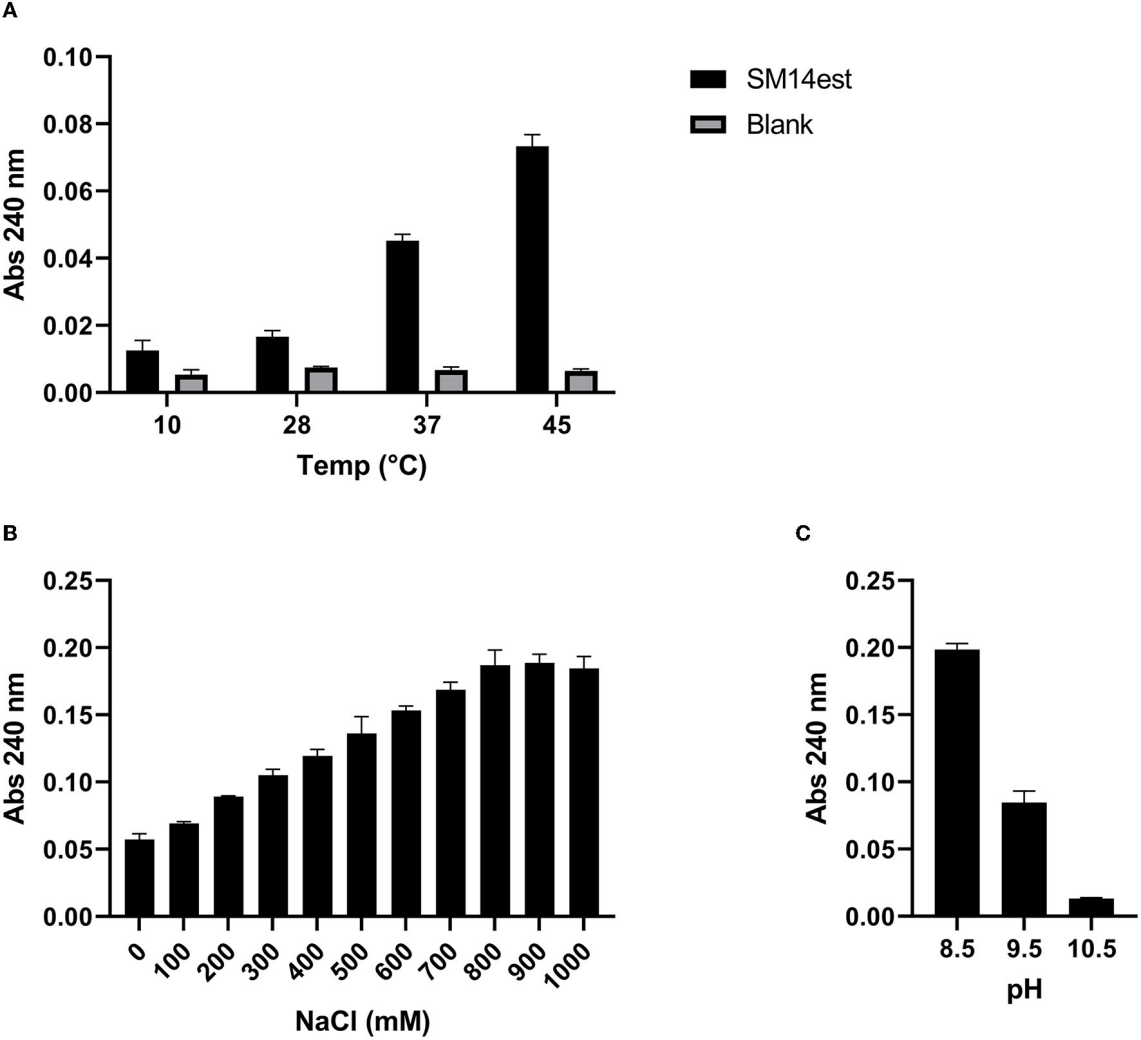
Effect of **(A)** temperature, **(B)** sodium chloride concentration, and **(C)** alkaline pH on the PET-hydrolyzing activity of SM14est. Reaction products were detected by absorbance at 240 nm following incubation of SM14est (0.5 μM) with PET (10 g L^−1^) in 50 mM sodium phosphate buffer, pH 8 except for **(C)** where 50 mM glycine buffer (with 500 mM NaCl added) was used. Incubations were set up as follows; **(A)** 1 h, 10–45°C and **(B, C)** 2 h, 45°C. Substrate blanks with no enzyme added served as a control.

The PET-hydrolyzing activity of the recombinant SM14est was assessed in the temperature range 10–45°C (Figure 3A), as well as in the presence of increasing concentrations of NaCl (Figure 3B), and in the alkaline pH range 8.5–10.5 (Figure 3C). SM14est was shown to hydrolyze PET, to varying extents, at a number of different temperatures; 10, 28, 37, and 45°C (Figure 3A). SM14est activity increased with the addition of NaCl, using concentrations up to 900 mM, at which point the effect appeared to plateau (Figure 3B). A marked decrease in the PET-hydrolyzing activity of SM14est was observed at pH 9.5 when compared to that measured at pH 8.5 and the enzyme performed poorly when tested at pH 10.5 (Figure 3C).

### 3.3. Characterization of SM14est activity toward PET

**Figure 4:**
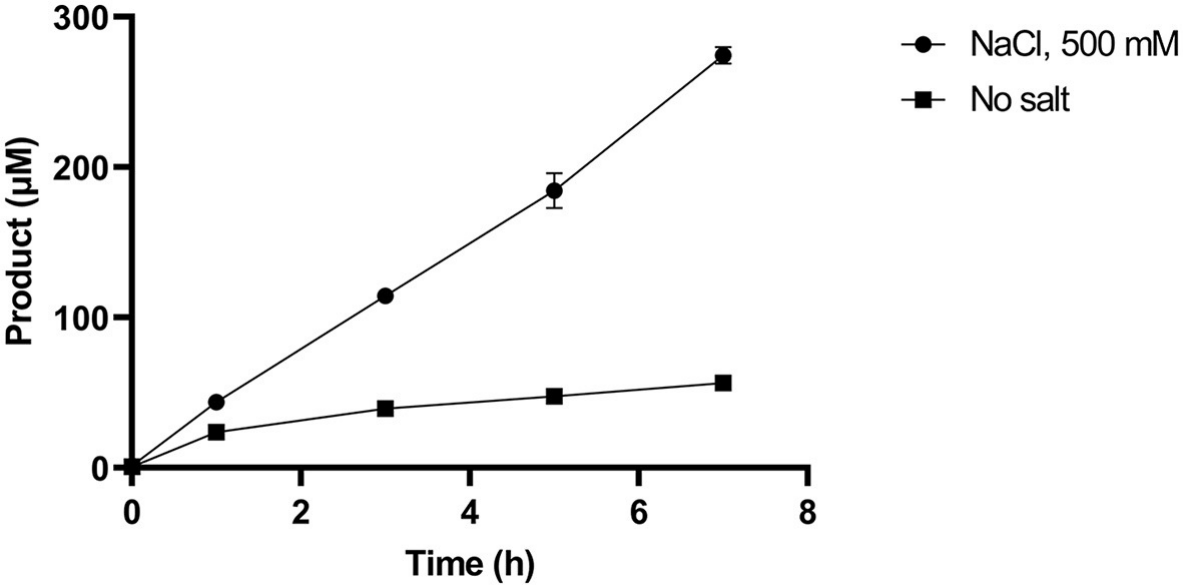
Product release over 7 h incubation of SM14est (0.5 μM) and PET (10 g L^−1^) in 50 mM sodium phosphate buffer, pH 8 with sodium chloride (500 mM) and without added salt at 45°C, quantified using a BHET standard curve.

Given that SM14est polyesterase activity increased at higher sodium chloride concentrations, the recombinant enzyme (0.5 μM) was subsequently assayed with PET (10 g L^−1^) as substrate over a 7 h period at pH 8 and 45°C, both with and without the addition of sodium chloride (500 mM; Figure 4). In absence of NaCl, reasonable levels of PET degradation were observed, reaching 56 μM product in the t_7_ sample. However, higher levels of activity were generated in the presence of NaCl, with 270 μM of product having accumulated in the equivalent t_7_ sample. The progress curves produced provide an insight into the initial rates of PET hydrolysis by SM14est, which were calculated to be 0.02 s^−1^ for the reaction with NaCl and in 0.004 s^−1^ in its absence (*R*^2^ = 0.9976 and 0.8959, respectively). This represents a 5-fold difference in the initial rate of product formation, and thus PET-hydrolyzing activity, when sodium chloride is included in the reaction vs. without the addition of salt.

**Figure 5:**
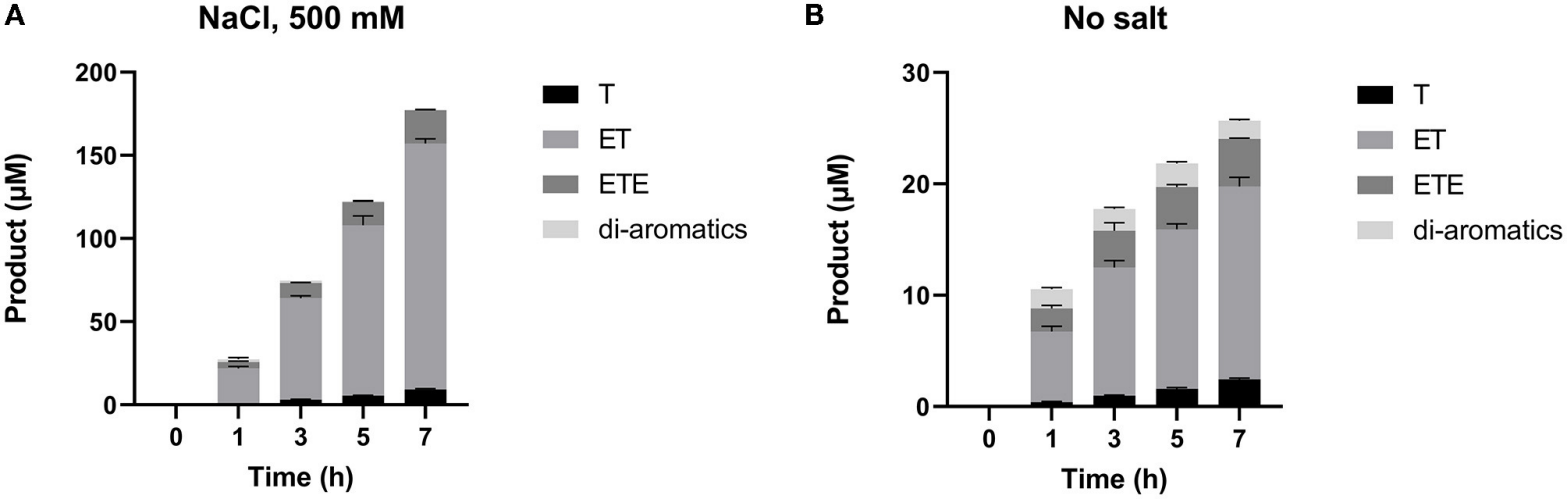
Individual products released during the 7 h time-course assay **(A)** with 500 mM NaCl added and **(B)** without additional salt, as detected by RP-HPLC. Mono-aromatic TPA, MHET, and BHET are represented by T, ET, and ETE, respectively (where T, terephthalic acid and E, ethylene glycol).

RP-HPLC-based analysis (Figure 5) was then performed to identify the reaction products generated during the 7-h time-course assay in Figure 4. This provides a breakdown of individual hydrolysis products, namely TPA, MHET, and BHET (shown here as T, ET, and ETE, respectively); as well as soluble di-aromatic oligomers which may include TET, TETE, ETETE. Tri-aromatic oligomers were not detected in any of the samples. It is clear from this analysis that SM14est displayed increased activity in the presence of sodium chloride, reflected in the generation of much higher quantities of PET degradation products. MHET was detected as the primary product of hydrolysis in both the presence (Figure 5A) and absence (Figure 5B) of sodium chloride; with minor secondary conversion of MHET to TPA, being observed with time.

## 4. Discussion

To date, known PET-hydrolyzing enzymes are derived from esterases, lipases, and cutinases, with thermostable proteins being prioritized since PET is most easily hydrolyzed when reaction conditions are set in accordance with the glass transition temperature (T_g_) of the plastic polymer (Lu et al., 2022). While thermotolerant enzymes have yielded promising results for the development of improved, bio-based recycling processes, it remains crucial to study enzymes that can efficiently degrade PET, among other plastics, under a variety of conditions to address the widespread issue of residual plastic that finds its way into natural ecosystems (Carr et al., 2022).

In the suspension-based assay employed for the characterization of SM14est, the aromatic ring structure present in MHET, BHET, and TPA enables UV-detection of these products at 240 nm, thus providing insight into the enzyme's PET hydrolase activity (Bååth et al., 2020). The substrate used here is of similar crystallinity to that of post-consumer PET waste (30–40 %), but exists in a powder form which is more readily depolymerized due to the particle size and available surface area (Gamerith et al., 2017). Pre-treatment by grinding and micronization are currently required for efficient enzymatic recycling of PET and, importantly, a large proportion of PET already exists as microparticles in the environment (Kawai, 2021).

While PET-hydrolyzing activity was observed at each temperature tested, SM14est performed best at 45°C (Figure 3A). Semi-crystalline PET is composed of both amorphous and crystalline regions and its T_g_ is 65–70°C in water (Kawai, 2021). The amorphous chains of PET have increased mobility around the T_g_, where they exist in a softened, rubber-like state (Thomsen et al., 2022). As reaction temperatures increase toward the T_g_, PET becomes more malleable and enzymes can better access its constituent chains, increasing the efficiency of hydrolysis (Figure 3A). SM14est has a T_m_ around 55°C, facilitating the high levels of activity observed at 45°C. By comparison, wildtype IsPETase, the mesophilic model PET hydrolase, has a lower T_m_ of 49°C, and displays optimal activity between 30 and 40°C (depending on the substrate) and loses activity after 1 h at temperatures above 40°C (Son et al., 2019; Kawai, 2021).

Based on the observed effect of increasing salt concentration, it appears the SM14est enzyme is halophilic and displays good salt tolerance (Figure 2B). The average salinity of seawater is commonly reported as being ~3.5% or 600 mM NaCl, and the general performance of the marine-derived SM14est seems to improve in accordance with this concentration range. The beneficial effect of sodium chloride on the activity of SM14est is further supported by the progress curves and product profiles generated in the presence and absence of salt (Figures 4, 5). In a previous study the halophilic PET hydrolase, PET6, from *Vibrio gazogenes*, was reported to exhibit a preference for very high salt concentrations, with optimum product release occurring between 1 and 1.5 M sodium chloride, when incubated at 30°C in PET-coated microtiter plates while using the well-studied IsPETase as a reference (Weigert et al., 2022). At a fixed concentration of sodium chloride (50 mM), PET6 exhibited distinct optimal activity between 45 and 50°C, while IsPETase displayed optimal activity at 30°C. In general, IsPETase displayed optimal activity at low salt concentrations, which rapidly decreases as ionic strength increases, while the opposite effect was observed for the PET6 enzyme. In the case of PET6, higher salt concentrations appeared to confer thermal stability, with a T_m_ increase from 49.8 to 57.7°C, and hence superior activity (Weigert et al., 2022).

**Figure 6:**
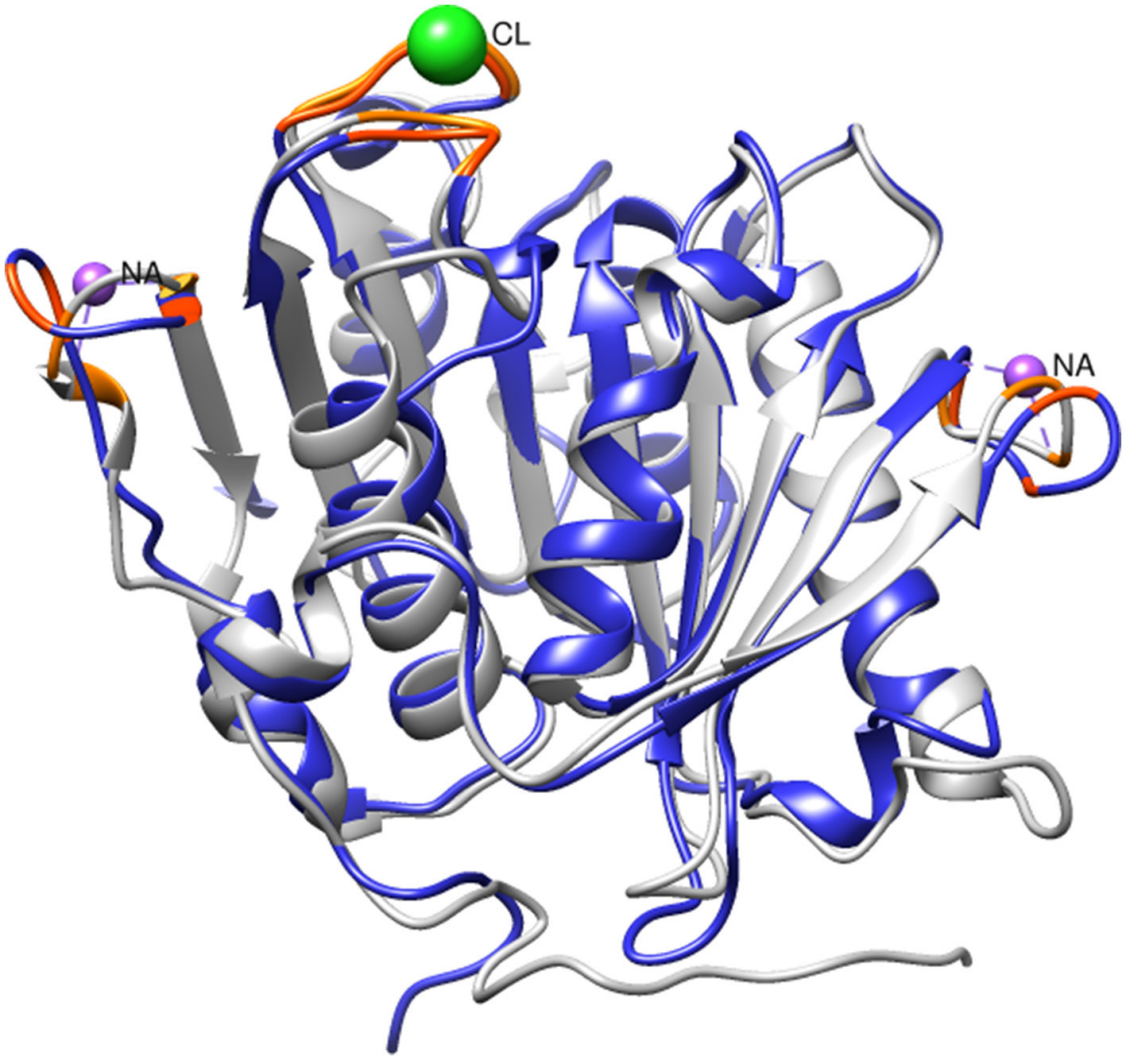
Superimposition of SM14est model (blue) onto PET6 structure (gray, PDB code: 7Z6B). Sodium and chloride ions (colored in purple and green, respectively) are shown at their PET6 binding sites, with relevant residues colored in orange. The corresponding residues in SM14est are colored in red-orange.

Given the observed increase in SM14est activity with the addition of sodium chloride, high salt conditions may elicit a stabilizing effect on SM14est, as seen with the halophilic PET6. Multiple sequence alignment of SM14est with PET6 demonstrates 75.89% similarity between both their protein sequences (Table 1), including conservation of the serine hydrolase catalytic triad (Ser163-Asp209-His241 in PET6; Ser131-Asp177-His209 in SM14est) and GXSXG motif (GWSMG in PET6; GHSMG in SM14est; Supplementary Figure 2A). The shared α/β fold arrangement, which was visualized by superimposing the Alpha-Fold model for SM14est onto the already resolved PET6 crystal structure (chain A), is well-conserved between these two enzymes (Figure 6). Structural analysis of PET6 previously revealed binding sites for monovalent ions, where one sodium ion interacts with Arg61, Ala63, and Phe65 residues, a second sodium ion is associated with Asp282, Ser284, and Val287, each occurring within a small loop via the amino acid backbone carbonyl groups (Weigert et al., 2022). In SM14est, Arg32, Ser35, and Phe38 correspond to the first ion site in PET6, while Gly248, Thr250, and Val253 are found in a loop around the second site (Figure 6). In PET6, a chloride ion sits in a shallow pocket comprised by Ala208, Asp209, Ala210, Val211, Ser240, His241, Phe242, which is similarly present in SM14est by Asn176, Asp177, Ala178, Ile179, Gly208, His209, and Phe210 (Figure 6; Weigert et al., 2022). This comparison indicates structural similarity between the two enzymes, supporting their potential adaptation to saline environments, which, when combined with the observed promotion of activity by sodium and chloride ions, provides further insights into marine-derived polyester hydrolases.

**Table 1:** Sequence similarities generated for SM14est against five functionally verified PET hydrolases.

**Enzyme**	**Origin**	**Identity (%)**	**Similarity (%)**	**References**
PHL7	*Thermoanaerobacter* sp.	46.13	79.41	Sonnendecker et al., 2022
IsPETase	*Ideonella sakaiensis*	41.03	78.21	Yoshida et al., 2016
LCC	Leaf-compost metagenome	38.51	76.40	Sulaiman et al., 2012
PET6	*Vibrio gazogenes*	34.40	75.89	Danso et al., 2018
PE-H	*Pseudomonas aestusnigri*	33.63	73.66	Bollinger et al., 2020
Ple629	*Marinobacter* sp.	32.26	72.87	Meyer Cifuentes et al., 2022

The Plastics-Active Enzymes Database (PAZy) has recently been established as a repository for enzymes with experimentally confirmed activities toward synthetic polymers, primarily those which act on PET (Buchholz et al., 2022). PET6, together with PE-H from *Pseudomonas aestusnigri*, and Ple629 from *Marinobacter* sp. are among the marine-derived PET hydrolases in the PAZy collection. Although SM14est shares over 70% amino acid sequence similarity with these three marine enzymes and between 32 and 34% sequence identity, it shares almost 80% similarity and just over 46% sequence identity with one of the top performing PET hydrolases, PHL7 (Table 1 and Supplementary Figure 2B). An engineered variant of PHL7 displays comparable activity to mutant LCC (ICCG), generating high levels of degradation products on a millimolar scale (Kawai et al., 2022). The identity and similarity percentages generated between SM14est and the benchmark enzymes LCC and IsPETase also ranked above those of the aforementioned marine examples, PET6, PE-H, and Ple629. This may indicate a potential for SM14est to represent an interesting “middle-ground” in the study of marine PET hydrolases, where its sea-sponge origin is complemented by its similarity to enzymes which have the highest known capacity for PET degradation. The product profiles obtained for SM14est by RP-HPLC (Figure 5) are comparable to those generated for thermophilic PET-hydrolyzing cutinases in a previous study, further evidencing the PET degradation ability of SM14est (Bååth et al., 2020).

**Figure 7:**
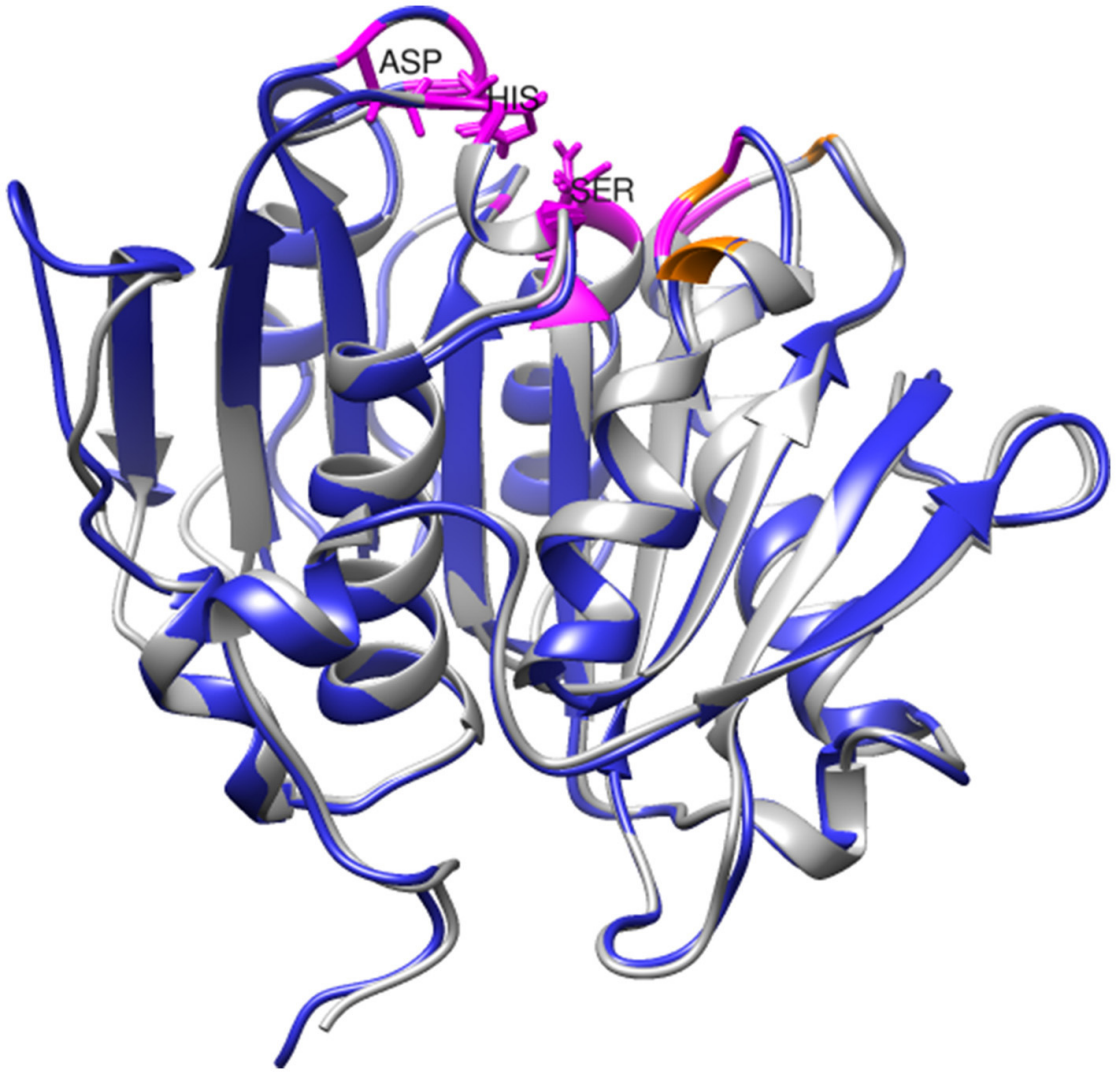
Superimposition of SM14est model (blue) onto PHL7 structure (gray, PDB code: 7NEI). Conserved active site residues are colored in magenta, distinguished from non-identical pocket residues, which are colored in orange. The catalytic triad residues (Ser131-Asp177-His-209) are labeled and shown in stick format.

Structural analysis and molecular docking experiments have previously been conducted to identify important amino acid residues in PHL7, particularly those contributing to its degradation efficiency and those with binding affinity for PET (Sonnendecker et al., 2022). The residues in the active site are highly conserved between SM14est and PHL7 (chain B; Figure 7). Within the substrate-binding cavity, Gly62, Thr64, Gln95, His130, Ser131, Met132, Trp156, Asp177, Ile179, Ala180, His209, Leu210, and Asn213 (catalytic triad residues underlined) are identical in SM14est and PHL7 (colored in magenta), with differences at just three positions; Tyr63, Thr69, and Tyr93 in SM14est correspond to Phe63, Ser69, and Leu93 in PHL7 (colored in orange; Figure 7). In PHL7, Leu210 was proposed as a particularly important residue for substrate binding and was experimentally verified to contribute to higher activity of PHL7 over the near-identical enzyme PHL3, which differs only by four amino acids overall. When this leucine was substituted for phenylalanine, which is found at this position in other type I PET hydrolases, the activity of PHL7 decreased to that of native PHL3 (Sonnendecker et al., 2022). The occurrence of leucine at position 210 in SM14est may have similar implications for PET binding and catalysis. Based on the PAZy-defined “PET hydrolase core domain,” Tyr63 in SM14est is likely to be an aromatic oxyanion hole residue involved in stabilizing the PET hydrolysis reaction intermediate, which occurs as tyrosine in 51% of homologous core domains (e.g., Tyr95 in LCC) and as phenylalanine in 46% (e.g., Phe63 in PHL7; Buchholz et al., 2022). In another study of PHL7, the replacement of phenylalanine with tyrosine at this position resulted in a variant with decreased hydrolytic activity but further investigation would be required to determine the role of Tyr63 in SM14est (Pfaff et al., 2022).

Since the identification of IsPETase in 2016, there has been an ongoing debate regarding the relevance of mesophilic PET hydrolases in the context of plastic waste management (Wallace et al., 2020; Kawai, 2021; Wei et al., 2022). Previously, it was highlighted that mesophilic PET hydrolases may be unsuitable for enzymatic PET recycling given the temperature requirements for efficient hydrolysis and furthermore, that attempts to improve the thermostability of IsPETase were contradictory to the original claims that this enzyme could enable PET hydrolysis under mild conditions (Kawai, 2021). Based on a study of LCC (ICCG) mutant and IsPETase molecular reaction mechanics, it has been suggested that the superior activity of known thermostable PET hydrolases is primarily due to the greater accessibility of PET polymer chains at higher temperatures, as opposed to their specific interaction with or affinity for the substrate (Boneta et al., 2021).

During the construction of the PAZy database, a PET hydrolase core domain was determined by Hidden Markov Model-based profiling and used to identify 2,930 homologs in the Lipase Engineering Database (LED), which was then assessed in terms of amino acid conservation (Buchholz et al., 2022). Using the LED PETase tool within PAZy, SM14est was aligned with IsPETase to compare positions that have previously been proposed to have structural or functional involvement in PET hydrolysis (Supplementary Figure 3). The isoleucine residue corresponding to Ile179 in SM14est has been implicated as a key residue for IsPETase substrate interaction. The tryptophan residue of subsite I, found at position 156 in SM14est, is referred to as the “wobbling tryptophan” in IsPETase since it is hypothesized to adopt three different conformations which confers increased flexibility within the substrate binding site. This effect is likely restricted in SM14est due to nearby His185 and Phe189 residues, but in other cases these have been successfully substituted for Ser and Ile (the neighboring residues in IsPETase) to enhance tryptophan mobility and thus, PET-hydrolytic activity (Aboelnga and Kalyaanamoorthy, 2022; Brott et al., 2022). Nonetheless, this tryptophan is believed to form stacking interactions with the aromatic component of PET and it is conserved in 77% of PET hydrolase core domain homologs (Buchholz et al., 2022). While many of the subsite I and II residues are equivalent in both IsPETase and SM14est, SM14est does not display the same extension in the second alpha-helix, another structural hallmark linked to superior IsPETase activity at ambient temperature (Sagong et al., 2021).

Recently, quantum mechanics/molecular mechanics (QM/MM) calculations have been employed for a more in-depth examination of the PET hydrolysis reaction pathway and the key structural features required for efficient catalysis (Jerves et al., 2021; Aboelnga and Kalyaanamoorthy, 2022). The IsPETase molecular features investigated, which enable the accommodation of PET in an advantageous bent conformation and promote tightened hydrogen bond interactions between catalytic triad residues, could prove useful in the rational engineering of SM14est. In particular, emphasis is placed on the role of the unique disulfide bond present near the IsPETase active site. Interestingly, the typical type I PET hydrolase disulfide bond (DS1) and the additional disulfide bond of IsPETase (DS2) are both absent from SM14est, which does not possess any disulfide bridge. Since we have now demonstrated that SM14est can use PET as a substrate, the introduction of DS1, DS2, or both could offer further insight into the role of such bonds in PET hydrolysis. Furthermore, the overall structure of SM14est could be modified using a machine learning-based approach to generate improved variants. A self-supervised, structure-based convolutional neural network called MutCompute was recently implemented to identify stabilizing mutations that resulted in FAST-PETase (functional, active, stable, and tolerant PETase^S121E/D186H/R224Q/N233K/R280A^), which emerged as an excellent candidate for *in situ* PET degradation when compared with wild-type IsPETase, previously engineered variants ThermoPETase and DuraPETase, as well as LCC and mutant LCC (ICCM), across a pH range of 6.5–8.0 and ambient temperatures of 30–40°C (Lu et al., 2022).

Halophilic enzymes are more likely to be resistant to proteolysis and denaturation by organic solvents and detergents, and often display a longer half-life that is particularly advantageous during the relatively slow process of plastic degradation (Atanasova et al., 2021). Plastic frequently contaminates marine and other salt-rich natural environments, together with high-salinity wastewaters, including those derived from agriculture, aquacultural, pharmaceutical, and chemical industries, that already pose a threat to surrounding ecosystems (Raju et al., 2018; Zhao et al., 2020; Atanasova et al., 2021). This highlights the need for bioremediation strategies that employ plastic-degrading bacteria and their enzymes; such approaches are under-represented in the field of PET hydrolases, where the primary goal to date has been to facilitate biological plastic recycling (Atanasova et al., 2021). Marine actinomycetes, including *Streptomyces* spp., that display halophilicity and halotolerance have previously been isolated from saline environments and shown to be both physiologically and metabolically distinct from their terrestrial counterparts (Almeida et al., 2019a). The work presented here on the PET-hydrolyzing activity of SM14est supports the need for the continued investigation of marine microorganisms and their enzymes, not only for the biological degradation of PET and other plastics, but also for the identification of industrially relevant biocatalysts.

## 5. Concluding remarks

While rapid advancements have been made in the study of PET hydrolases, the problem of plastic waste is multi-faceted and requires a diverse suite of microbes and enzymes to enable both environmentally sound, closed-loop recycling as well as biological remediation of plastic-contaminated sites. Here, the hydrolytic activity of the marine polyesterase SM14est is characterized using PET as a substrate. SM14est can hydrolyze PET at a range of temperatures, most efficiently at 45°C, but even as low as 10°C, which may be indicative of an ability to act on PET in its native environment. Furthermore, SM14est displayed a strong preference for pH 8 and high salt concentrations, each aligning with the typical conditions found in ocean waters. Following structural protein analyses, SM14est was shown to share similarities as well as key differences with other well-characterized PET hydrolases, including those from marine sources. Although there is a possibility that, as a result of microbial evolution, the enzymatic machinery in marine bacteria could become adapted to degrade the increasing levels of plastics and microplastics to which they are being exposed, we suspect that it is the promiscuous nature of hydrolase enzymes such as SM14est that primarily facilitates the acceptance of an unnatural substrate like PET. Nonetheless, given that marine-specific actinomycetes have been studied and shown to populate a variety of habitats, which as of yet remain a largely neglected resource as of yet, this work on the sea sponge-derived *Streptomyces* enzyme SM14est provides further incentive to explore different types of marine ecosystems as a source of novel enzymes with potentially interesting biotechnological applications.

## Data availability statement

The original contributions presented in the study are included in the article/Supplementary material, further inquiries can be directed to the corresponding author.

## Author contributions

CC designed the experiments with the support of MK, BP, KJ, PW, and AD. Experimental work was performed by CC, BP, SS, and KC. CC, SS, and KC analyzed the data. KJ, PW, and AD contributed reagents, materials, and analysis tools. CC and AD wrote the manuscript. PW, DC, and AD supervised the study. All authors approved the final manuscript.
