# Supplementary material for: Purification and biochemical characterization of SM14est, a PET-hydrolyzing enzyme from the marine sponge-derived Streptomyces sp. SM14
Source: Front Microbiol. 2023 May 12;14:1170880. doi: 10.3389/fmicb.2023.1170880 (PMC10213408; doi:10.3389/fmicb.2023.1170880)
Supplement: Supplementary file 1 [file Presentation_1.pdf]

Supplementary Material

Purification and Biochemical Characterization of SM14est, a PET-Hydrolyzing Enzyme from the Marine Sponge-Derived *Streptomyces* sp. SM14

Clodagh M. Carr[1,2], Malene B. Keller[3], Bijoya Paul[3,4], Sune W. Schubert[3], Kristine S.R. Clausen[3], Kenneth Jensen[4], David J. Clarke[1,6], Peter Westh[3], Alan D.W. Dobson[1,2,5*]

*** Correspondence:** Alan D. W. Dobson: a.dobson@ucc.ie

1 Supplementary Figures

Figure S1. AlphaFold model of SM14est three-dimensional protein structure (A), where central beta-sheet is colored in cyan and alpha-helices colored are in orange. The complementary quality data is shown (B), with Predicted Aligned Error (PAE) matrices, sequence coverage plot, and per-residue confidence measure (pLDDT) included for the top five ranked models.

A

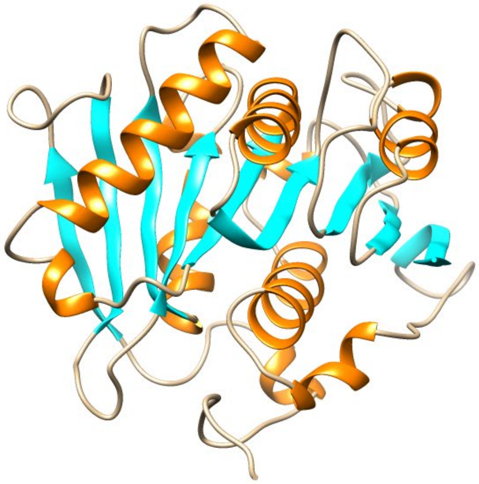

B

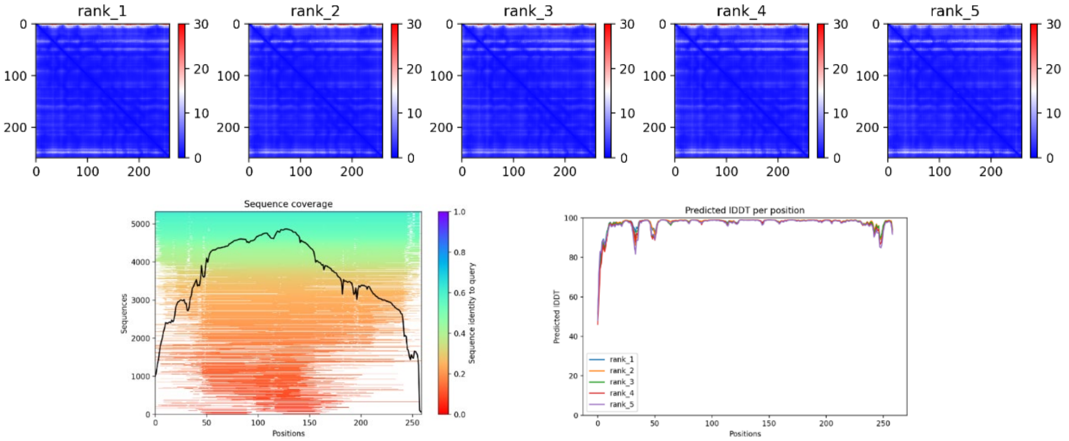

Figure S2. Amino acid sequence alignments of SM14est with (A) PET6, and (B) PHL7.

A

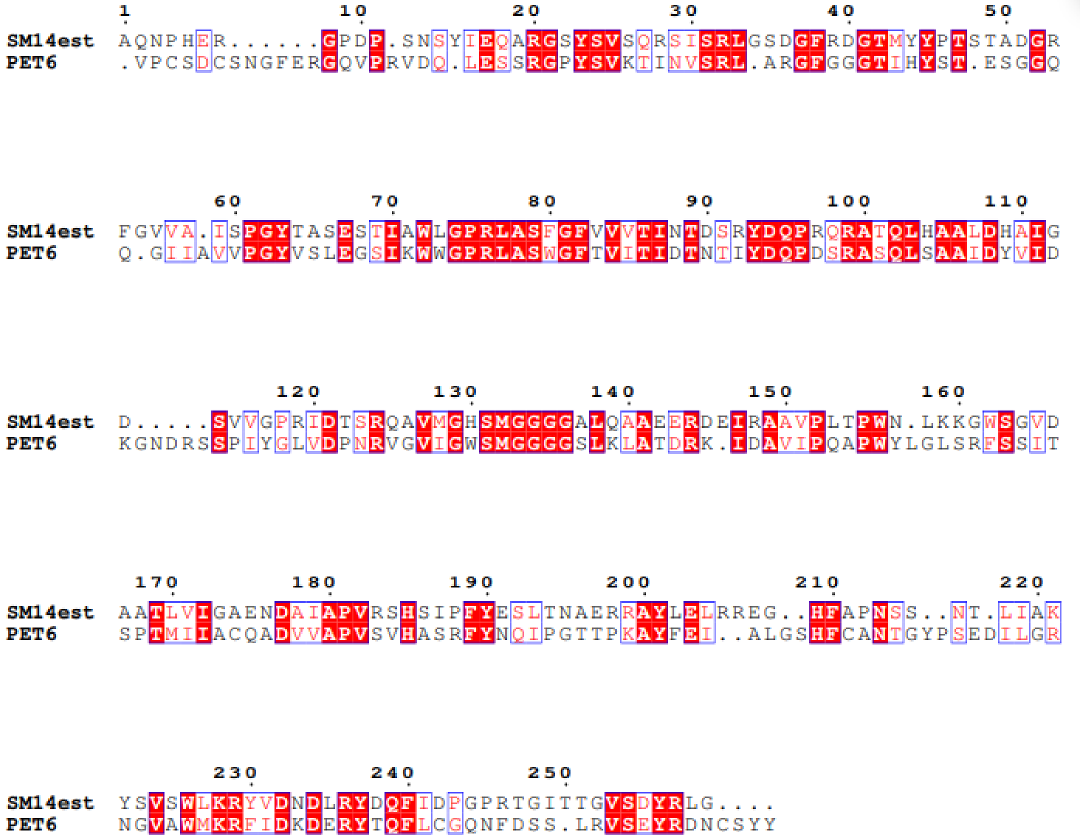

B

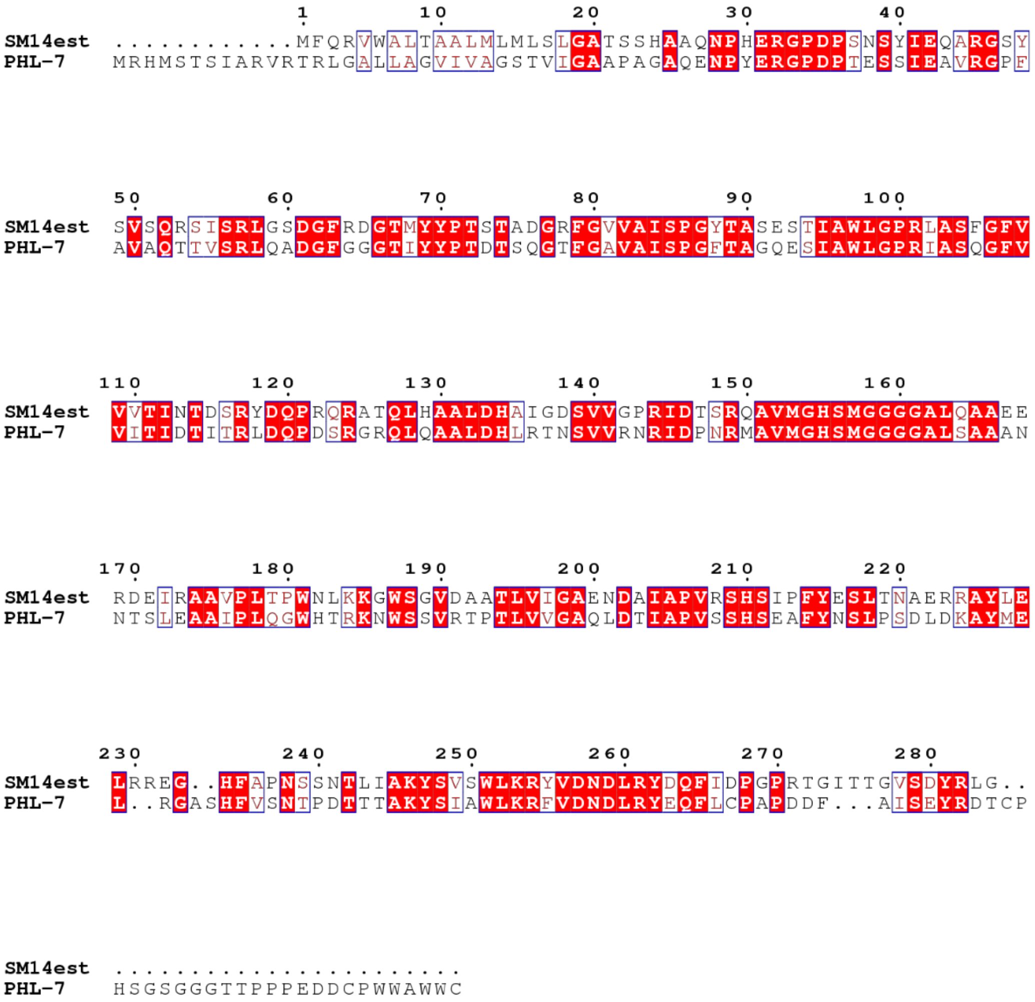

Figure S3. PAZy-LED output, generated by aligning SM14est (query) sequence with IsPETase (reference) sequence.

```
AQNPHERGPD PSNSYIEQAR GSYSVSQRSI SRLGSDGFRD GTMYYPTSTA  50
DGRFGVVAIS PGYTASESTI AWLGPRLASF GFVVVTINTD SRYDQPRQRA  100
TQLHAALDHA IGDSVVGPRI DTSRQAVMGH SMGGGGALQA AEERDEIRAA  150
VPLTPWNLKK GWSGVDAATL VIGAENDAIA PVRSHSIPFY ESLTNAERRA  200
YLELRREGHF APNSSNTLIA KYSVSWLKRY VDNDLRYDQF IDPGPRTGIT  250
TGVSDYRLG
```

alignment of your query and the reference sequences

```
query:      ---------- ------A--- --------QN PHERGPDPSN SYIEQARGSY  23
reference:  MNFPRASRLM QAAVLGGLMA VSAAATAQTN PYARGPNPTA ASLEASAGPF  50

query:      SVSQRSISRL GSDGFRDGTM YYPTSTADGR FGVVAISPGY TASESTIAWL  73
reference:  TVRSFTVS-R P-SGYGAGTV YYPTNA-GGT VGAIAIVPGY TARQSSIKWW  97

query:      GPRLASFGFV VVTINTDSRY DQPRQRATQL HAALDHAIGD S-----VVGP  118
reference:  GPRLASHGFV VITIDTNSTL DQPSSRSSQQ MAALRQVASL NGTSSSPIYG  147

query:      RIDTSRQAVM GHSMGGGGAL QAAEERDEIR AAVPLTPWNL KKGWSGVDAA  168
reference:  KVDTARMGVM GWSMGGGGSL ISAANNPSLK AAAPQAPWDS STNFSSVTVP  197

query:      TLVIGAENDA IAPVRSHSIP FYESLTNAER RAYLELRREG HFAPNSSN--  216
reference:  TLIFACENDS IAPVNSSALP IYDSM-SRNA KQFLEINGGS HSCANSGNSN  246

query:      -TLIAKYSVS WLKRYVDNDL RYDQFIDPGP RTGITTGVSD YR-----LG
reference:  QALIGKKGVA WMKRFMDNDT RYSTFACENP --NS-TRVSD FRTANCS--
```

Residue in SM14est	Residue in IsPETase	Feature
Tyr63	Tyr87	Subsite I, aromatic clamp/oxyanion hole
Thr64	Thr88	Subsite II
Ala65	Ala89	Subsite II
Gln95	Gln119	Subsite I
-	Gly139	Extension in alpha-helix 2
-	Ser141	Extension in alpha-helix 2
His130	Trp159	Subsite II
Ser131	Ser160	Catalytic triad, nucleophilic serine
Met132	Met161	Subsite I, oxyanion hole
Trp156	Trp185	Subsite I, aromatic clamp
Asp177	Asp206	Catalytic triad
Ile179	Ile208	Substrate interaction
His209	His237	Catalytic triad
Phe210	Ser238	Subsite II
Asn213	Asn241	Subsite II
Ser214	Ser242	Extended loop
-	Gln247	Extended loop
